# Identification of Gene Expression Signatures for Phenotype-Specific Drug Targeting of Cardiac Fibrosis

**DOI:** 10.3390/ijms24087461

**Published:** 2023-04-18

**Authors:** Dominika Lukovic, Ena Hasimbegovic, Johannes Winkler, Julia Mester-Tonczar, Katrin Müller-Zlabinger, Emilie Han, Andreas Spannbauer, Denise Traxler-Weidenauer, Jutta Bergler-Klein, Noemi Pavo, Georg Goliasch, Sandor Batkai, Thomas Thum, Faiez Zannad, Mariann Gyöngyösi

**Affiliations:** 1 Department of Internal Medicine II, Division of Cardiology, Medical University of Vienna, 1090 Vienna, Austria; ena.hasimbegovic@meduniwien.ac.at (E.H.); johannes.winkler@univie.ac.at (J.W.); julia.mester-tonczar@meduniwien.ac.at (J.M.-T.); katrin.zlabinger@meduniwien.ac.at (K.M.-Z.); emilie.han@meduniwien.ac.at (E.H.); andreas.spannbauer@meduniwien.ac.at (A.S.); denise.traxler-weidenauer@meduniwien.ac.at (D.T.-W.); jutta.bergler-klein@meduniwien.ac.at (J.B.-K.); noemi.pavo@meduniwien.ac.at (N.P.);; 2 Hannover Medical School Institute of Molecular and Translational Therapeutic Strategies (IMTTS), 30625 Hannover, Germanythum.thomas@mh-hannover.de (T.T.); 3 Fraunhofer Institute for Toxicology and Experimental Medicine (ITEM), 30625 Hannover, Germany; 4 Inserm Clinical Investigation Centre, Université de Lorraine, CHU, 54052 Nancy, France; f.zannad@chru-nancy.fr

**Keywords:** porcine model, myocardial fibrosis, transcriptomics, iLINCS

## Abstract

We have designed translational animal models to investigate cardiac profibrotic gene signatures. Domestic pigs were treated with cardiotoxic drugs (doxorubicin, DOX, n = 5 or Myocet^®^, MYO, n = 5) to induce replacement fibrosis via cardiotoxicity. Reactive interstitial fibrosis was triggered by LV pressure overload by artificial isthmus stenosis with stepwise developing myocardial hypertrophy and final fibrosis (Hyper, n = 3) or by LV volume overload in the adverse remodeled LV after myocardial infarction (RemoLV, n = 3). Sham interventions served as controls and healthy animals (Control, n = 3) served as a reference in sequencing study. Myocardial samples from the LV of each group were subjected to RNA sequencing. RNA-seq analysis revealed a clear distinction between the transcriptomes of myocardial fibrosis (MF) models. Cardiotoxic drugs activated the TNF-alpha and adrenergic signaling pathways. Pressure or volume overload led to the activation of FoxO pathway. Significant upregulation of pathway components enabled the identification of potential drug candidates used for the treatment of heart failure, such as ACE inhibitors, ARB, ß-blockers, statins and diuretics specific to the distinct MF models. We identified candidate drugs in the groups of channel blockers, thiostrepton that targets the FOXM1-regulated ACE conversion to ACE2, tyrosine kinases or peroxisome proliferator-activated receptor inhibitors. Our study identified different gene targets involved in the development of distinct preclinical MF protocols enabling tailoring expression signature-based approach for the treatment of MF.

## 1. Introduction

Cardiac injury, pressure and volume overload [1], myocardial infarction [1] and treatment with cardiotoxic cytostatic drugs [2] are pathogenetic events that all result in the development of myocardial fibrosis (MF) leading to cardiac remodeling and heart failure (HF). MF is the cumulative result of dynamic changes manifesting on the molecular, cellular and interstitial levels that represent compensatory mechanisms in response to myocardial injury. Depending on the pathological stimuli affecting the myocardium, distinct remodeling cascades have been described, involving loss of cardiomyocytes (CMs), CMs hypertrophy and the development of MF. Despite the distinct molecular signals involved in the histopathology of MF, the essential common denominator of the cellular alternations is the signaling pathways involved in cell death/cell survival, inflammation, oxidative stress signaling and regulation of ion channels [3]. The main consequences of such molecular and structural modifications generate the functional, structural and electrical substrate for the progression of HF and sudden cardiac death [4].

Treatments aimed at reversing MF processes and targeting its molecular activators have become the main objective of HF therapy. To date, several pre-clinical studies have analyzed the mechanisms responsible for the development of HF, enabling the identification of several drugs with beneficial effects in terms of attenuating the progression of HF [1,5,6,7,8].

Research on MF has thus far been hampered by a limited access to human myocardial samples. Therefore, the development of meaningful in vivo translational animal models of MF is indispensable for the understanding of potentially treatable molecular mechanisms.

Here, we present multiple translational porcine models of different MF etiologies. We induced replacement fibrosis by the treatment of domestic pigs with doxorubicin drugs which possess known cardiotoxic effects. Diffuse replacement fibrosis occurs by exposure of the heart to toxic agents, local or systemic inflammatory processes or during scar formation after myocardial infarction and is associated with cell damage or necrosis, leading to the degradation of normal tissue, which is replaced by newly formed extracellular matrix fragments [9].

Furthermore, we have developed an animal model of pressure overload-induced with myocardial hypertrophy and a volume overload model induced by ischemic cardiomyopathy. Diffuse interstitial MF, which is characterized by an excessive deposition of collagen fibers through the entire myocardium [10], was observed in both models. Infiltrative diffuse interstitial fibrosis emerges from the deposition of insolvable proteins such as amyloid or glycosphingolipids [11].

We hypothesized that distinct models of MF might exhibit relatively similar but also unique gene expression signature profiles, enabling the targeting of particular genes that play substantial roles in the progression of HF.

In the current study, we performed a secondary analysis of transcriptomic data obtained via a next-generation sequencing (NGS) approach. We retrieved previously published data [2] describing the differential gene expression in anthracycline-treated animals which demonstrated diffuse MF. Together with bulk RNA sequencing data retrieved from the pig models that exhibit progressive cardiac hypertrophy with MF and a myocardial infarction model with focal MF, we compared global gene expression signatures of these experimental models against untreated animals and connected the signatures with drugs perturbagens present in the iLINCS database in order to search for novel treatment approaches.

## 2. Results

### 2.1. Induction of CR Was Accompanied by the Development of MF

We have previously demonstrated that treatment with the cytostatic anthracycline anti-cancer drugs, doxorubicin and Myocet induces the development of severe MF in the LV of pigs, as shown by cMRI imaging and histology [2]. We observed substantial fibrosis and myocyte hypertrophy in the LV tissue in pigs with percutaneous artificial aortic stenosis [1]. Catheter-based reperfused anterior wall myocardial infarction in mid-LAD increased myocardial fibrosis in the remote area with signs of adverse ventricular remodeling [12]. Histological analyses confirmed pronounced MF and deposition of collagen (red color) in all fibrosis models (Supplementary Figure S1).

### 2.2. Analysis and Summary of RNA-Seq Data Quality

Induced MF of animal models treated with cardiotoxic reagents (DOX and MYO groups) or in animals undergoing percutaneous coronary intervention (PCI; RemoLV and Hyper groups) induced molecular changes translated into differential gene expression. We performed bulk RNA-seq in order to analyze global transcriptome changes of LV myocardial tissues in experimental models of MF. The analysis generated a total number of sequenced reads ranging from 12 to 29 million pairs (Supplementary Figure S2A), of which 87.3.8–91.5% of the reads were uniquely mapped to the Sus Scrofa genome (Sus scrofa 11.1) assembly using the STAR-alignment tool. The percentage of genomic alignment was similar between all groups, thus suggesting that there were no obvious detectable biases on the sequence data (Supplementary Figure S2B).

### 2.3. Global Gene Expression Changes in the Myocardium in Distinct Myocardial Fibrosis Models

**Figure 1:**
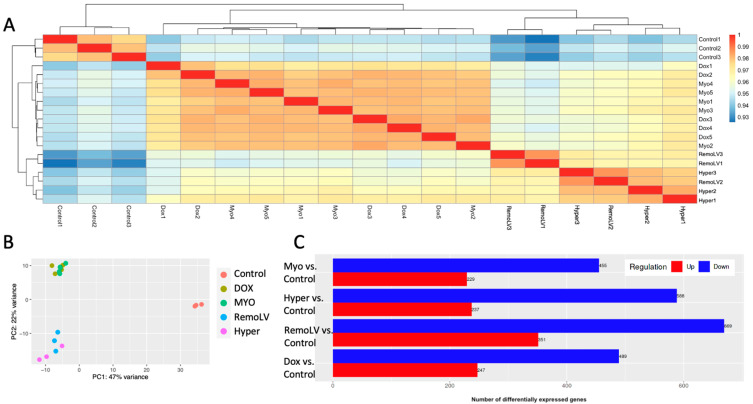
Global gene expression changes in MF models. Unsupervised two-dimensional hierarchical clustering of samples based on gene expression level (**A**). Different colors in principal component analysis indicate different treatment models (**B**). Bars representing the number of up- and down-regulated genes in all treatment groups compared to control with adjusted cut-offs: FDR = 0.05, LFC = ±2 (**C**).

The HTseq-DESeq2 pipeline was used to generate the counts and to estimate their abundance following the preparation of gene-level count-matrices (Figure 1A). The expression patterns of the mRNA, as quantified by RNA-seq, in the remodeled myocardial tissue were distinct from normal tissue as evidenced by a principal component analysis (Figure 1B). The sample-to-sample distance heat map showed an acceptable degree of similarity among biological replicates, demonstrating the reliability of RNA-seq data and being a prerequisite for differential expression analysis (Figure 1A). Unsupervised hierarchical clustering clearly separated the samples according to the treatment procedure into three clusters: control group, groups treated with cardiotoxic reagents (DOX, MYO) and groups with animals undergoing percutaneous coronary intervention (RemoLV, Hyper).

The largest number of differentially expressed genes (DEGs) generated RemoLV vs. Control group, followed by Hyper, MYO and DOX groups with an absolute log2 fold change ≥ 2 and a false discovery rate (FDR) of ≤0.05 (Figure 1C). There were 1020 DEGs for the RemoLV group (↑up-regulated 351, ↓down-regulated 669), 825 for the Hyper vs. Control group (↑237, ↓588), 736 for the DOX vs. Control group (↑247, ↓489) and 684 for the MYO vs. Control group (↑229, ↓455). A total of 338 DEGs were common in all treatment groups as compared to the Control group.

### 2.4. Comparison of Transcriptional Profiles Indicate Pathological Phenotype of CR

**Figure 2:**
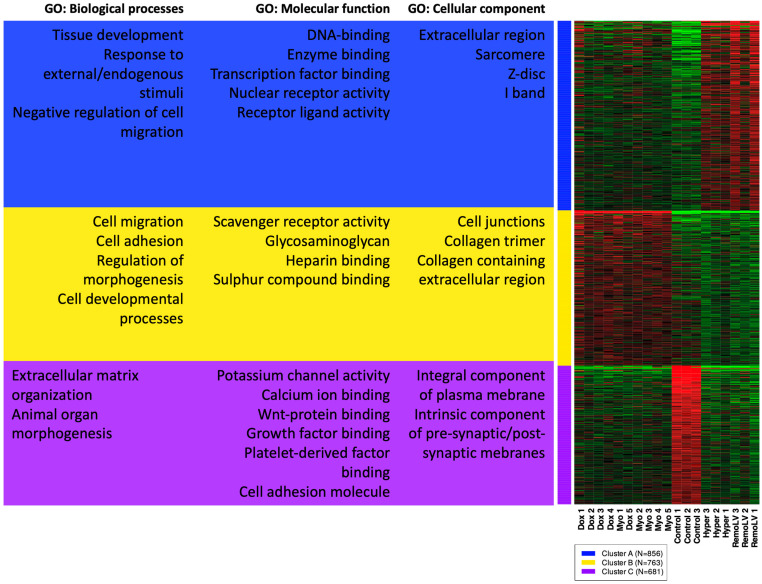
Heatmap of k-means clustering of genes with subsequent gene ontology (GO) enrichment analysis. Unsupervised gene clustering ordered genes into groups based on the expression pattern across all samples. 2000 top-ranked expressed genes were used for a GO enrichment analysis for each gene cluster. The figure shows the most associated GO cellular components, biological processes and molecular functions for each cluster of genes. Green and red colors denote highly and weakly expressed genes, respectively.

The heatmap of k-means clustering showed that the gene expression profile of the most variable genes in the DOX and MYO groups were similar. Likewise, similar gene expression patterns were observed in RemoLV and Hyper groups (Figure 2).

The most significantly enriched GO terms in the category cellular component (CC) showed that mechanistic components of myocytes and contractile apparatus were upregulated only in RemoLV and Hyper groups (Cluster A), whereas upregulation of collagens and extracellular matrix-related genes were observed mostly in DOX and MYO groups (Figure 2, Cluster B).

**Figure 3:**
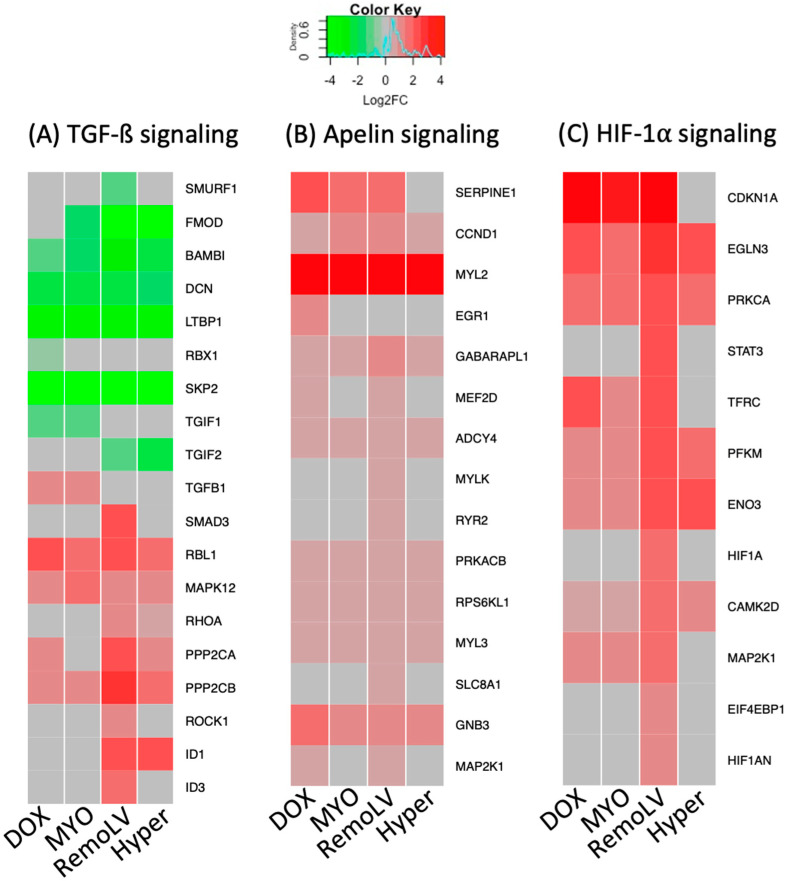
The heatmaps of differentially regulated genes in regulated pathways across all models of myocardial remodeling. The heat maps show the expression of genes compared to untreated control in the transforming growth factor beta (TGF-ß) signaling pathway (**A**), apelin signaling pathway (**B**), and hypoxia-inducible factor 1-alpha (HIF-1 alpha) signaling (**C**) in all treatment groups. Red = upregulation; green = downregulation; gray = non-significant changes. The treatment groups DOX (N = 5), MYO (N = 5), RemoLV (N = 3) and Hyper (N = 3) are relative to control animals (N = 3). (Significance for *p* < 0.05, moderated t-statistics adjusted for multiple testing).

Furthermore, upregulated genes clustered in the GO term of biological processes (BP) were mostly involved in the response to endogenous stimuli and tissue development in Groups RemoLV and Hyper, while upregulation of genes associated with tissue and anatomical structure development, cell migration and adhesion and related to abnormal heart tissue development and morphology were detected in groups DOX and MYO (Figure 3, Cluster A, B). These results indicate that cardiotoxicity-induced CR exerts a distinct expression profile in terms of mechano-structural alternations as compared to pressure and volume overload models of MF.

Downregulation of genes coding for proteins with metallopeptidase activity and for extracellular matrix structural constituent in Cluster C (molecular function) in all treatment groups coincided with collagen deposition in the extracellular space (Figure 2, Cluster C) in all experimental models as compared to control animals. Clusters and a complete list of assigned genes in individual GO terms are included in Supplementary File S1.

### 2.5. Pathway Activation Differs between the Experimental Models

To further dissect the molecular process and, in particular, the upstream signaling pathways in the individual models, we performed an extensive comparison of gene set functional enrichment (GSEA) in pre-ranked mode (fGSEA) and gene networks for all experimental models compared to the Control group (Supplementary File S2). Two experimental settings, cardiotoxicity- (DOX, MYO) and pressure- and volume-overload-induced (RemoLV, Hyper) cardiac fibrosis resulted in the activation of distinct molecular changes within the LV.

As the most prominent pathway involved in the development of MF, TGF-ß signaling pathway was de-regulated across all treatment groups. The gene expression of TGF-ß signaling inhibitors (*SMURF1, FMOD, BAMBI, DCN, LTBP1, RBX1, SKP2, TFGIF1, TGIF2*) was downregulated, whereas pathway components, transcription factors and activators were upregulated (Figure 3A). The gene components of apelin pathways with known cardioprotective effect were overexpressed both in cardiotoxic-induced samples (DOX, MYO) and in RemoLV and Hyper groups (Figure 3B). Another common upregulated pathway among all treatment groups was HIF-1 alpha signaling pathway with the strongest upregulation in the RemoLV group (Figure 3C).

**Figure 4:**
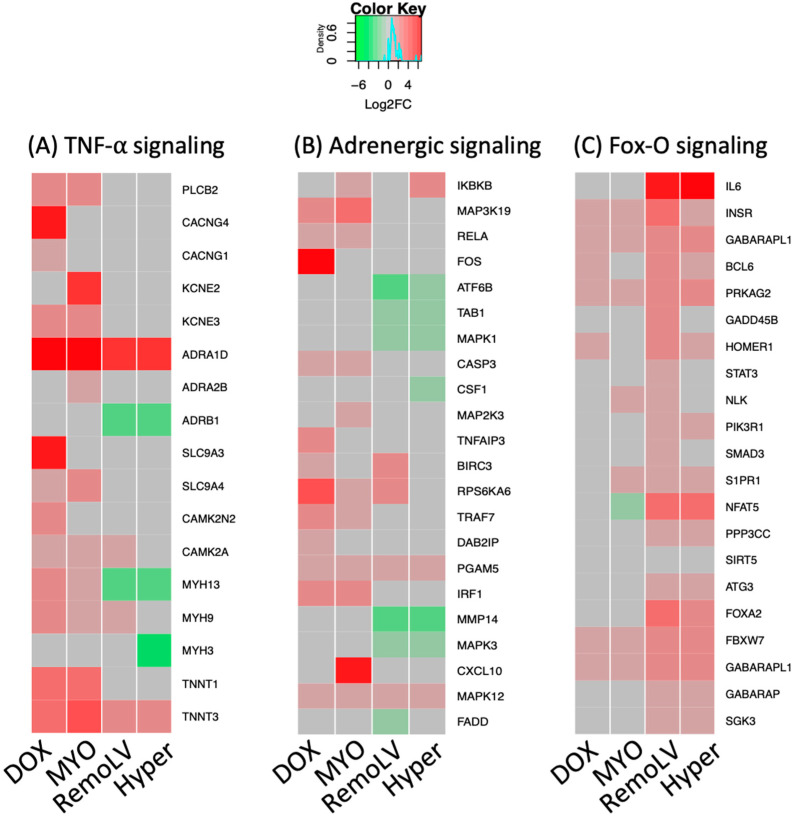
The heat maps of DEGs in significantly regulated pathways in specific models of MF. The heat maps show the expression of genes compared to untreated control in the tumor necrosis factor-alpha (TNF-alpha) signaling pathway (**A**); adrenergic signaling pathway (**B**); and Forkhead box protein-O (Fox-O) signaling (**C**) in all treatment groups. Red = upregulation; green = downregulation; gray = non-significant changes. The treatment groups DOX (N = 5), MYO (N = 5), RemoLV (N = 3) and Hyper (N = 3) are relative to control animals (N = 3). (Significance for *p* < 0.05, moderated t-statistics adjusted for multiple testing).

Aside from the activation of these signaling pathways, which were observed in all experimental models, we identified pathways with a distinct signature between cardiotoxic drug-induced CR and myocardial remodeling caused by volume or pressure overload, respectively. Application of anthracycline anti-cancer drugs activated the TNF-alpha and adrenergic signaling pathways (Figure 4A) in cardiomyocytes (Figure 4B). Several significantly over-expressed genes in DOX and MYO groups were either not significantly regulated or downregulated in RemoLV and Hyper groups, such as *TNNT1* (coding skeletal TroponinT), *ADRB1* (adrenoreceptor beta1), *CASP3* (apoptosis signaling), *FOS* (cell transformation) or *KNE3* (potassium ion and voltage-gated channel gene). This suggests that inflammatory and adrenergic processes play a pivotal role in the development of MF induced by cardiotoxic agents. In contrast, fibrosis resulting from pressure- and volume-overload-induced heart failure did not show activation of inflammatory processes in the myocardium.

The induction of remodeling processes by pressure or volume overload, which exhibited the activation of FoxO pathway components and are known to play an important role in cardiac hypertrophy, was documented. We found simultaneous upregulation of HIF-1α and transcription factors of FoxO signaling pathways in the RemoLV model. Similarly, pressure overload resulted in CR by an increased expression of genes involved in FoxO signaling and PI3K signaling. The FoxO pathway was activated in RemoLV and Hyper groups but not in the groups of anti-cancer drugs (Figure 4C).

### 2.6. Transcriptomics Signature of Animal Models of MF Related to Drug Prediction

In order to identify potential drugs, which may reverse the expression signature profile of MF models with accompanied developed cardiac remodeling and heart failure, we subjected the significantly dysregulated genes of each treatment model to the iLINCS database. We selected the drugs that exhibited significant concordance with transcriptional signature in the particular MF model and divided them into groups based on the common gene target.

**Figure 5:**
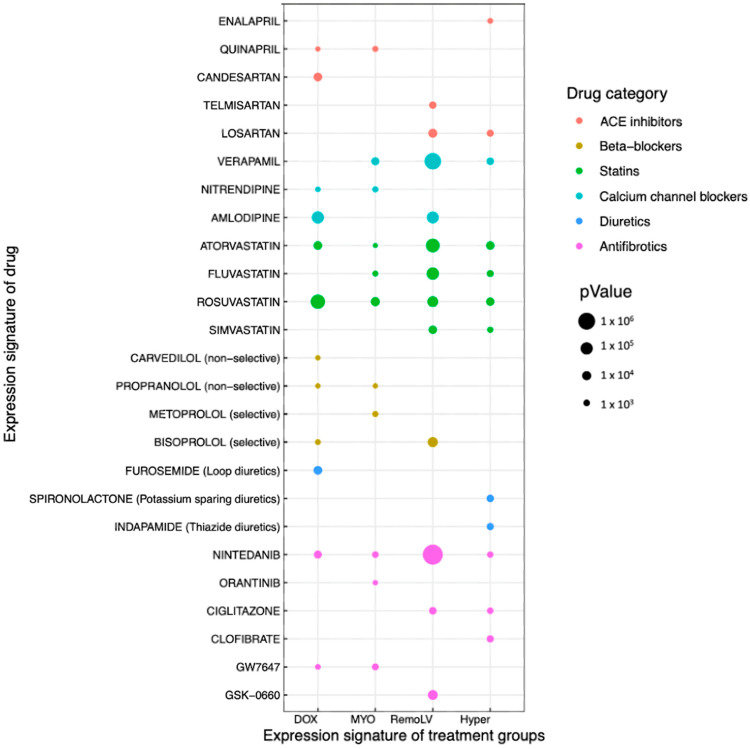
Drug prediction based on the gene expression signature datasets. Bubble plot lists of selected significant chemical perturbagens (predicted drugs) based on the gene expression signature datasets of DOX (N = 5), MYO (N = 5), RemoLV (N = 3) and Hyper (N = 3) groups. Selected perturbagens reflect (Pearson’s correlation coefficient) the gene expression signatures of MF models. The size of the dot represents *p*-value. ARB, angiotensin II receptor blocker; ACE, angiotensin converting enzyme.

We found that the group of renin angiotensin aldosterone system (RAAS) antagonists would reverse the transcriptional signatures found in the CR models. Despite the common gene targets angiotensin II receptor type 1 and angiotensin converting enzyme (*ACE*) (Angiotensin converting enzyme receptor-1; *AGTR1*), some inhibitors were associated only with the DOX and MYO groups (candesartan, irbesartan, quinapril) and others (enalapril, telmisartan, losartan) had a correlation in RemoLV and Hyper treatment groups (Figure 5).

We identified the drugs targeting calcium, potassium channels (verapamil, amlodipine, nitrendipine) and statins inhibiting 3-hydroxy-3-methylglutaryl-CoA reductase (HMGCR; atorvastatin, fluvastatin, rosuvastatin) across all models of CR. This is in concordance with DGA that showed upregulated genes coding the subunits of calcium and potassium channels across all models of CR (Figure 5*)*.

Since the pathway analysis showed the upregulation of adrenergic signaling in DOX and MYO groups (Figure 4B), beta blockers—competitive antagonist of adrenergic receptors (*ADRA*, *ADRB*)—were selected for further analysis. We found most of the perturbagens with a significant concordance of gene expression signature in DOX and MYO groups (carvedilol, propranolol, metoprolol, bisoprolol) and single perturbagen in RemoLV (bisoprolol) group (Figure 5). Analysis of expressed genes showed significant upregulation of main interaction partners of the particular perturbagens. Mineralocorticoid receptor antagonists exhibited concordance with the RemoLV (eplerenone) and Hyper (spironolactone) groups. Loop diuretic furosemide exhibited concordance with the DOX group and thiazide-like diuretic with the Hyper group (indapamide). We identified the group of antifibrotic drugs that antagonize peroxisome proliferator-activated receptors (PPAR) and threonine kinase receptors (TKRs) used either in clinical (nintenadib, orantinib, ciglitazone, clofibrate) or experimental settings (2-CHLORO-5-NITRO-N-4-PYRIDINYLBENZAMIDE, GW7646, GSK-0660) to decrease fibrosis.

Based on the GO enrichment analysis, we focused on FOX-o signaling molecular pathway and its transcription factors that might reveal novel drug targets. We searched for expression signatures involving FOX-o signaling gene components in the iLINCS database. We found a single drug thiostrepton that targets the *FOXM1*-regulated angiotensin-converting enzyme (ACE) switch. Analysis of the FOXM-1-associated genes revealed a significant regulation of *FOXM1* interaction partners (*GFM, EFTUD2, RPL11, RPL10, EIF5B, EEF2*) in RemoLV and Hyper groups (Supplementary Figure S3). This suggests that thiostrepton might exhibit a beneficial effect in the reduction of the CR effect via ACE regulation, especially in the pressure- and volume-overload-induced model.

## 3. Discussion

MF is the common final stretch of a number of chronic and acute cardiac conditions. The development of efficient treatment approaches is hampered by the insufficient assessment of origin and stage of MF. In the current study, we applied multiple translational porcine models in order to investigate distinct types of MF. Pressure overload with gradual increase of aortic stenosis (Hyper), volume overload induced by occluded ischemia/reperfusion (RemoLV), and anthracycline-induced cardiotoxicity (DOX, MYO) revealed complex etiologies, all of which resulted in the development of myocardial fibrosis. In addition, pigs represent a model with a 95% extent of genetic sequence homology with humans supporting the use of porcine models for accurately capturing the transcriptomic signature of myocardial fibrosis [13]. Pigs are a scientifically acceptable intermediate species between rodents and humans to model several pathophysiological conditions and cardiovascular functions relevant to humans.

Unsupervised hierarchical clustering revealed a significant overlap between volume and pressure overload MF. Although the liposomal doxorubicin formulation (Myocet) has lower cardiotoxicity than non-liposomal doxorubicin, their RNAseq signatures were relatively similar and differed substantially from the other MF models. We successfully identified specific molecular signaling pathways for each type of MF, allowing identification of distinct mechanistic pathways involved in remodeling processes and selection of potential novel treatment options.

Doxorubicin and Myocet are cytotoxic chemotherapeutic agents with known cardiotoxic properties [14,15]. We previously confirmed an induction of MF in a porcine model treated with either Doxorubicin or Myocet, with differences in interferon-related DNA damage resistance, and a milder toxicity profile of Myocet [2]. In the current analysis, we focused on the specific gene signature of established animal models, and observed an upregulated expression of genes involved in TNF-alpha and adrenergic signaling in both the DOX and MYO groups compared to the control group. This confirms the fact that cardiotoxicity-induced MF exhibits stronger apoptotic and stress responses compared to pressure- or volume-overload-induced MF.

The RemoLV and Hyper groups both exhibited the enrichment of pathways of significantly upregulated genes, including the HIF-1α pathway and transcription factors of FoxO signaling in cardiomyocytes. The FoxO transcription factors are regulated by upstream signaling molecules such as PI3K, AMP-kinase or SIRT genes, and regulate the downstream transcription of proteins such as calcineurin, muscle RING finger1, muscle atrophy F-box, pyruvate dehydrogenase kinase 4 and coactivator-1α [16]. This has a direct impact on the regulation of protein synthesis, apoptosis and autophagy, finally leading to structural changes of the myocardium, such as MF [17,18]. We found a significantly upregulated expression of both upstream and downstream interaction partners of FoxO molecular effectors in these MF models, confirming the important role of FoxO signaling in the development and progression of MF.

These shared and distinct molecular mechanisms offer potential for novel etiology-based molecularly targeted therapeutic strategies. By comparing the transcriptome signatures of the experimental models of MF with the chemical perturbation signatures in the iLINCS database, we have identified potential therapeutic agents that may be optimal and adapted to the disease etiology among drugs that are already in use for treating heart failure. We identified the perturbagen drugs (Candesartan, Telmisartan, Losartan) that inhibit the angiotensin 1 (*AGTR-1*) receptor. Candesartan and enalapril have already been tested in registered clinical trials and were found to significantly ameliorate MF in hypertensive patients [19]. The combination of RNA-seq and correlation of transcriptomics data with drug signature profiles in our animal models reassert the beneficial effect of RAS inhibitors in MF. Based on the identified RNA signatures, the potential of individual RAS antagonists differ between the MF models, with some agents being more favorable for treating cardiotoxic drug-induced MF, while others are more beneficial for volume- or pressure-induced MF. In addition, a DEG analysis revealed a significant overexpression of the neprilysin gene in DOX, RemoLV and Hyper groups. Therefore, the neprilysin inhibitor sacubitril might be a good therapeutic option in cardiotoxicity and pressure- and volume-overload-induced MF. The distinct correlations of drugs within the same class and our in vivo models of MF might indicate that the comparison of bulk sequencing data with transcriptomic data of cell lines exposed in vitro to a variety of perturbing agents might introduce some degree of discrepancy in a transcriptome-based drug prediction model.

Furthermore, we identified several perturbagen drugs in the class of statins, reflecting gene expression signatures in all types of MF models (atorvastatin, fluvastatin, rosuvastatin, simvastatin) with developed MF. Interestingly, all statins inhibit 3-hydroxymethylglutaryl-CoA reductase as a main mechanism, but the drug prediction model resulted in a difference between statins and ARB blockers by being useful in one, but not in the other remodeling models. Experimental and clinical data indicate that lipid lowering mediated by statins in resident cardiomyocytes and fibroblasts inhibits adverse remodeling processes in ischemic and non-ischemic settings [20]. Although there is no known anti-fibrotic effect with statins, statins inhibit inflammatory processes and can therefore reduce chronic inflammation-based tissue fibrosis. These findings and systems-biology-level information retrieved from our gene expression data warrant further research in order to ascertain the beneficial effects of statins on MF which result in ventricular remodeling.

Mineralocortioicid receptor antagonists have been associated with decreased serum markers of fibrillar collagens [21]. In our gene expression signature dataset, we identified mineralocortioicid receptor antagonists (spironolactone and eplerenone), indicating antifibrotic properties of these drugs.

Indapamide and furosemide seem to potentially interfere with MF mechanisms. Several clinical trials have already confirmed the antifibrotic effects of furosemide [22], the recent LIVE study confirmed that indapamide leads to a significant decrease in LV mass in patients with LV hypertrophy [23].

Our data showed an upregulation of adrenergic signaling, with an increased expression of genes encoding adrenergic receptors and pathway components in the DOX and MYO groups. We found either non-selective (carvedilol, propranolol) or selective ß-blockers (metoprolol, bisoprolol) across all animal experimental models. Beneficial effects of ß-blockers on cardiomyocyte hypertrophy and MF have been observed in rats [24]. However, clinical studies failed to provide unambiguous evidence that ß-blockers affect MF [25,26].

In addition, we identified the class of anti-fibrotic perturbagens inhibiting receptor tyrosine kinases (Nintedanib, Orantinib) and peroxisome proliferator-activated receptors (PPAR) inhibitors (Ciglitazone). Recent studies confirmed that both RTKs and PPAR inhibitor drugs identified in our analysis have shown significant cardioprotective effects and benefits which lead to fibrosis regression [27,28].

A further major mechanistic finding of our study is the confirmed regulation of FoxO signaling in the RemoLV and Hyper groups. Analysis of drug matrix signatures targeting the component of FoxO signaling (transcription factor *FOXM1*) retrieved a single result: thiostrepton. A study by Yang et al. found that thiostrepton induces a conversion of ACE to ACE2 in rats [6]. Inhibition of *FOXM1* expression by miR-204 or thiostrepton leads to reduced vascular remodeling in pulmonary arterial hypertension [29]. Additionally, potential benefits of inhibiting *FOXM1* via thiostrepton treatment have been demonstrated in vitro and in a rat model with interstitial fibrosis [30]. Our data, combined with the findings of these studies indicate that FOXM1 might be a promising novel therapeutic target in the treatment of pressure- or volume-overload MF accompanied with adverse cardiac remodeling.

Our gene expression analysis of three models with distinct MF etiologies, combined with an analysis of the molecular signature of drugs in the iLINCS database, is a promising approach for identifying biologically relevant candidate therapeutics with the potential to ameliorate MF.

## 4. Materials and Methods

### 4.1. Animal Study Design

Twenty-seven pigs (*Sus scrofa*, female, large whites) were included in the study. Five animals received either doxorubicin (Pfizer Inc. New York, NY, USA) (DOX, n = 5) or liposomal-encapsulated doxorubicin, Myocet (TEVA Ratiopharm, Ulm, Germany) (MYO, n = 5), and five animals received a vehicle and served as controls (n = 5). Five pigs underwent reperfused myocardial infarction (RemoLV, n = 5) or an induced artificial aortic isthmus stenosis (Hyper, n = 7). The sham-operated control group served as the control group (n = 5). The detailed experimental protocols have previously been published [1,2].

Briefly, animals in groups DOX and MYO received either doxorubicin (DOX) or liposomal–doxorubicin citrate complex (Myocet^®^, MYO) in 3 cycles (Day 1, 22, 43) of cytostatic treatment in doses equivalent to human treatment regimens (60 mg/m^2^ body surface area). The DOX and MYO experiments had to be prematurely terminated after 3 months (FUP) due to progressively deteriorating health conditions of the animals.

Domestic pigs in the group RemoLV were sedated by subcutaneous administration of 12 mg/kg ketamine hydrochloride, 1 mg/kg xylazine and 0.04 mg/kg atropine after overnight fasting. After endotracheal intubation, the anesthesia was supplemented with 1.5–2.5 vol% isoflurane, 1.6–1.8 vol% O_2_ and 0.5 vol% N_2_O. A 6F introduction sheath (Terumo Medical Corporation, Somerset, NJ, USA) was inserted into the right femoral artery. Following the administration of 200 IU/kg of heparin, 6F right coronary guiding catheters were introduced into the left and right coronary ostium to perform selective angiography of the left and right coronary arteries. Catheter-based reperfused anterior wall myocardial infarction was induced by the inflation of a coronary angioplasty balloon (2.75 mm diameter, 15 mm length; Maverick, Boston Natick, MA, USA) at 5 atm for 90 min in the middle part of the left anterior descending the coronary artery. Reperfusion was initiated via balloon deflation. The animals were allowed to recover from the anesthesia. FUP investigations and myocardial tissue sampling were performed at the sixth month.

For pressure-overload-induced myocardial fibrosis (Hyper), a bare metal stent (9 mm of diameter, 20 mm of length, Cordis S.M.A.R.T. CONTROL, Cordis, Fremont, CA, USA) was implanted in the descending thoracic aorta of the juvenile pigs. Natural enlargement of thoracic aorta by growing animals reaching the diameter of 1.8 cm resulted in induced artificial aortic thoracic stenosis by a constant 9 mm stent. The developing artificial aortic isthmus stenosis resulted in the gradual development of a myocardial hypertrophy and diffuse myocardial fibrosis during the sixth month of FUP.

After the predefined FUP, the animals were euthanized under the continuous deep anesthesia (1.5–2.5 vol% isoflurane, 1.6–1.8 vol% O_2_, and 0.5 vol% N_2_O) using an intravenous dose of heparin (10,000 U) and 10 mL of saturated KCl (10%). Immediately after humanely euthanizing the animals, the hearts were explanted and myocardial samples from the anterior wall in groups DOX, MYO and Hyper were collected, while remote posterior wall myocardial area from the remodeled LV was excised in group RemoLV. The hearts were not perfused with phosphate-buffered saline in order to avoid prolonged sample manipulation duration at room temperature, which would have affected RNA quality.

**Figure 6:**
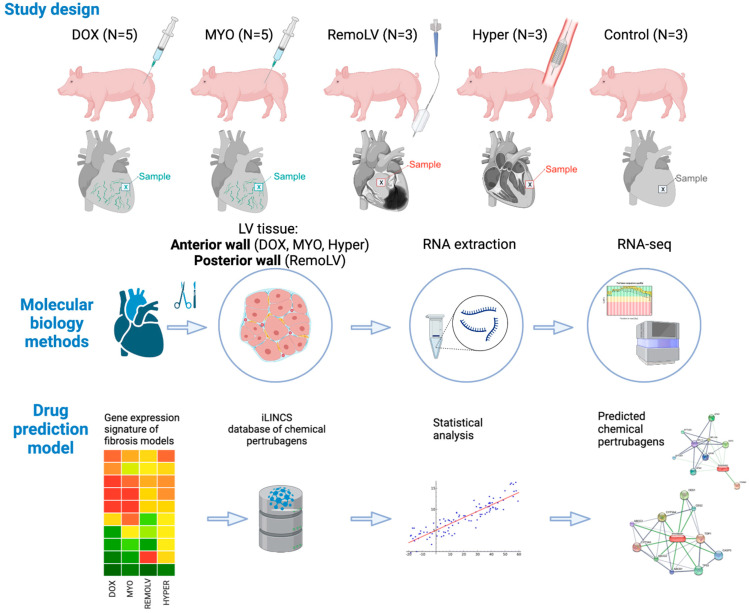
Study design and applied molecular biology methods. The study population comprises 5 groups of pigs. DOX animals (N = 5) received doxorubicin and MYO animal (N = 5) Myocet^®^ infusions in doses equivalent to human treatment regimens. Catheter-based reperfused anterior wall myocardial infarction was induced by balloon inflation in mid-LAD, and induced adverse remodeling of the enlarged left ventricle (LV), resulting in volume-overload myocardial fibrosis of the posterior non-ischemic wall (RemoLV, N = 3). Artificial aortic isthmus stenosis was designed to study pressure overload-induced myocardial remodeling (Hyper, N = 3). Sham interventions served as control (Control, N = 3). After a pre-defined follow-up (FUP), the hearts were explanted and subjected to RNA expression analysis following RNA-seq bioinformatics pipeline and drug prediction model. Graphical elements in both panels were created with BioRender.com.

Nineteen animals were included in the sequencing study (DOX, n = 5; MYO, n = 5; RemoLV, n = 3; Hyper, n = 3; Control, n = 3). The control group in the sequencing study is made up of untreated animals of the same age (Figure 6).

### 4.2. RNA Isolation

Myocardial tissue samples (~30 mg) of the left ventricle (anterior wall from groups HYPER, DOX, MYO and Control, and non-ischemic posterior myocardial wall of group RemoLV) were washed with PBS, minced into small pieces and transferred into cryotubes containing 1.2 mL RNAlater^®^ (Ambion, Austin, TX, USA). The specimens were homogenized using the Precellys lysing kit CK28 beads (VWR, Vienna, Austria) in Qiazol lysis buffer (Qiagen, Hilden, Germany) and total RNA was isolated using the RNeasy^®^ tissue kit (Qiagen, Hilden, Germany).

### 4.3. RNA Sequencing

The isolated total RNA samples were subjected to mRNA deep sequencing using the Illumina (San Diego, CA, USA) platform by the Next Generation Sequencing Facility at Vienna BioCenter Core Facility (VBCF, Vienna, Austria). The NEBNext Poly(A) mRNA Magnetic Isolation Module (New England Biolabs, Ipswich, MA, USA) was utilized for mRNA fragmentation and enrichment. Fragmented and primed mRNA was reverse transcribed to cDNA. The cDNA library was synthesized and enriched using the NEBNext Ultra Directional RNA library kit (New England Biolabs, Ipswich, MA, USA). Sequencing was performed on the HiSeq 2500 platform (Illumina, San Diego, CA, USA) to a mean depth of 15–20 million paired-end reads per sample. After demultiplexing, the quality control was performed by FastQC following mapping to the Sus scrofa genome (Sscrofa 11.1) using STAR-alignment tool (STAR 2.7.8a) [31] on a MacOS BigSur computer (Version 11.4, Processor: 2,4 GHz 8-Core Intel Core i9, Memory: 64 GB 2667 MHz DDR4).

Finally, mapped reads were counted into the Ensembl Sscrofa 11.1 gene model using HTSeq Python package (Python3 Version 3.9, HTSeq-Count Version 0.11.1) [32], applying parameters: “-m union stranded = revers”. Differential expression analysis was performed with DESeq2 package (Version 3.16) [33] in R Studio (Version 1.4.1106).

Raw reads were transformed based on library size using DESeq2 package with subsequent rlog transformation. Statistically significantly deregulated genes were determined by generalized linear models fitted to the corresponding contrast (each treatment versus control group). The estimated coefficients and standard errors for all contrasts were used for calculation of moderated t-statistics, moderated F-statistics, and the log-odds of differential expression by empirical Bayes shrinkage of the standard errors toward a common value.

Rlog transformed data were used for hierarchical clustering of the top 1000 differentially expressed genes, utilizing heatmap.2 function. The data were centered by subtracting the average expression level for each gene. The distance matrix is 1 − *r*, where *r* is Pearson’s correlation coefficient. The average linkage was used.

Principal component analysis (PCA) was performed with rlog transformed data using web-based tool iDEP [34].

Clustering of genes based on their expression pattern across all sample groups was performed using k-means clustering methods [34]. Cluster of genes were subjected to the enrichment analysis based on cellular component, molecular function and disease gene ontology. Then, student’s t-test was used to compare the scores observed in a group of genes. The *p*-values were corrected for multiple testing using false discovery rate (FDR). Gene set enrichment analysis was conducted in the pre-ranked mode using algorithm in fgsea package [35].

Gene expression data were visualized either with heatmap2. function in gplots package (v3.1.1, R studio) or utilizing KEGG pathway diagrams in PathView [36].

### 4.4. Drug Prediction Using Reverse Transcriptomics Signature Approach

In order to identify drugs which may reverse the gene expression signature acquired from differential gene expression analyses of MF models compared to untreated control animals, we utilized the data from the Library of Integrated Network-based Cellular Signatures (LINCS) (http://www.ilincs.org/ilincs/, accessed on 20 January 2023). As input for the drug prediction, the DEGs were used with log2FC, adj.*p*-values (adj.*p*-value < 0.05) and assigned known gene symbol.

We compared the global transcriptional signature of each animal model with available LINCS chemical perturbagen signatures. Each comparison generated hundreds of matched perturbagen signatures. To standardize our analysis, we selected only chemical perturbagens with negative Pearson’s correlation coefficients and ordered the list of drugs based on *p*-values. The generated list of all chemical perturbagens in particular groups is included in Supplementary Materials (Supplementary File S3, available upon reasonable request). The prediction of association between drug and particular gene expression signature of treatment group is based on the inverse correlation, presuming that each drug would abolish the given treatment-specific expression signature. Next, we grouped the chemical perturbagens based on the common inhibition of gene targets in the group of ARB/ACE inhibitors, beta-blockers, statins, calcium channel blockers and diuretics. Recurring target drugs were plotted using ggplot2 package in R studio (v3.3.3).

### 4.5. Histology and Picrosirius Staining

For histological sections, LV myocardial tissue samples were stored in 5 % formalin, embedded in paraffin and cut to 4–5 µm slices followed by PicroSirius red staining. The color coding is as follows: myocytes—yellow, fibrosis—red. Histological images were acquired on an Olympus IX83 microscope and images were analyzed with CellSens software (Olympus, Tokyo, Japan).

### 4.6. Statistics

For differences between treatment and control groups, DESeq2 packages used a Wald test: the shrunken estimate of LFC is divided by its standard error. The Wald test’s *p*-values that pass independent filtering were adjusted for multiple testing using the procedure of Benjamini and Hochberg corrections [33]. A difference was considered statistically significant at FDR < 0.05. Statistical analysis and graph preparations were performed using R studio and iDEP web-based programs.

## 5. Conclusions

In summary, we provide evidence that cardiac injury, including MF, induced by cardiotoxic drugs, cardiac pressure or volume overload resulted in overlapping but also in distinct gene expression signature profiles. We identified significantly dysregulated pathways involved in the development of cardiac injury and ventricular remodeling that may be differently targeted by individual treatment groups. Additionally, we present a novel approach of drug prediction by comparing the robust expression data from sequencing analysis and pre-computed drug expression signature in iLINCS database, providing an abundant source of information regarding the molecular reactions to drug candidates in order to help identify relevant candidate therapeutics for cardiac repair.

## 6. Study Limitations

To achieve a comprehensive understanding of mechanisms involved in the development of MF and its therapeutic targets, we focused on bioinformatics analysis in this study. Additional experimental evidence would be necessary to confirm the therapeutic value of the proposed agents.

There are several limitations of this study that should be acknowledged.

First, we compared the RNA-seq data in different animal models of chronic cardiac structural changes leading to distinct types of cardiac remodeling and multiple degrees of heart failure. The most common feature of these experiments were the findings of MF in histology and cardiac MRI. Moreover, several MF-related gene expression signatures were common in these experiments, suggesting at least partially similar pathophysiological processes leading to MF. Since no specific antifibrotic therapy exists, we have focused on the MF. RNA extraction from LV and library preparation protocols were compatible in all analyzed groups in order to mitigate potential batch effects; all samples were taken at a phase in which development of MF was evident.

Second, we only performed a single time-point analysis based on the resulting development of LV remodeling. In order to clarify the dynamic changes of gene targets, it will be necessary to conduct further studies with different time-points. Such experiments are usually conducted on small animal models, including pharmacokinetic and pharmacodynamic studies, mainly due to financial reasons before starting human-like large animal studies, and therefore lie beyond the scope of current investigations.

Third, we compared the data derived from bulk RNA sequencing of the pig LV tissue with the gene expression signatures generated in distinct cell culture cell lines, as data in the iLINCS database are available for all drug targets in the cell lines only. Our approach has identified candidate repurposable drugs for the small molecules in the iLINCS repository that may attenuate MF. The potential drug candidates are: (A) currently approved for the use in humans, (B) have demonstrated anti-fibrotic effect in pre-clinical and clinical studies and (C) need to be investigated to assess direct anti-fibrotic effects.

Considering the limited number of animals, we decided on using female domestic pigs in order to exclude sex-related gene expression differences. For our sequencing study, we included a uniform control group with healthy animals of the same age as the experimental animals. The inclusion of a control group specific to each treatment would have potentially resulted in more robust findings.

Our results were obtained in a porcine model, and the translational potential of our findings to human patients should be investigated in further studies.

Our results were obtained in a porcine model and drug predictions reflected the captured pathological phenotype and ‘reverse’ drug signature in the iLINCS database. Further exploration should be performed to determine whether our findings can be translated to human conditions.

## Data Availability

The data discussed in this publication have been deposited in NCBI’s Gene Expression Omnibus [37] and are accessible through GEO Series accession number GSE197049.

## Supplementary Materials

The following supporting information can be downloaded at: https://www.mdpi.com/article/10.3390/ijms24087461/s1.
