# Supplementary material for: Identification of Gene Expression Signatures for Phenotype-Specific Drug Targeting of Cardiac Fibrosis
Source: Int J Mol Sci. 2023 Apr 18;24(8):7461. doi: 10.3390/ijms24087461 (PMC10139067; doi:10.3390/ijms24087461)
Supplement: Supplementary file 1 [file ijms-24-07461-s001.zip › ijms-2324655-figures.pdf]

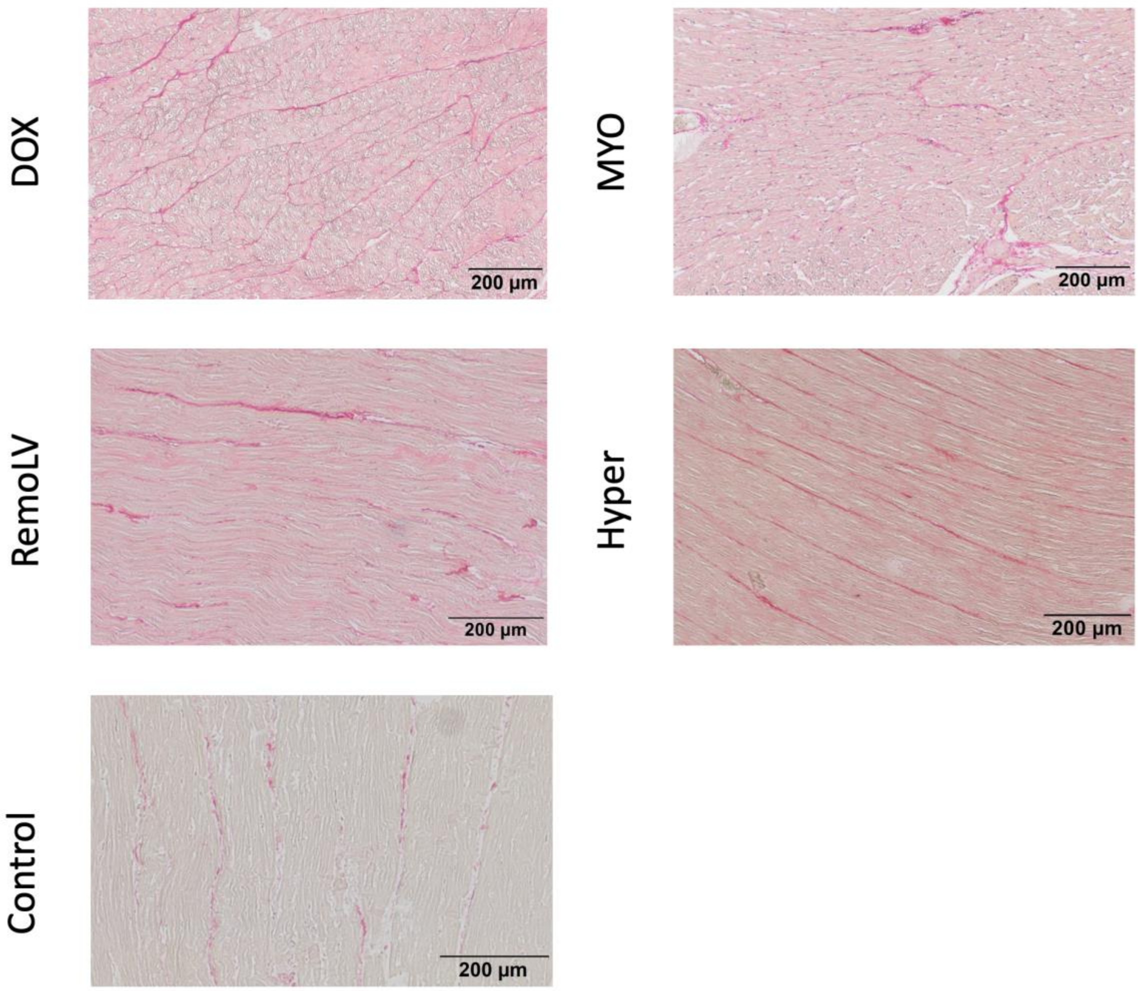

Supplementary figure S1. PicroSirius Red staining of the left ventricle (400x).

Histological fibrosis assessment of the anterior myocardium after doxorubicin (DOX) and Myocet treatment (MYO), and interstitial area of the posterior myocardium in pigs with volume-overload induced myocardial fibrosis (RemoLV). Red color shows pronounced myocardial fibrosis.

A

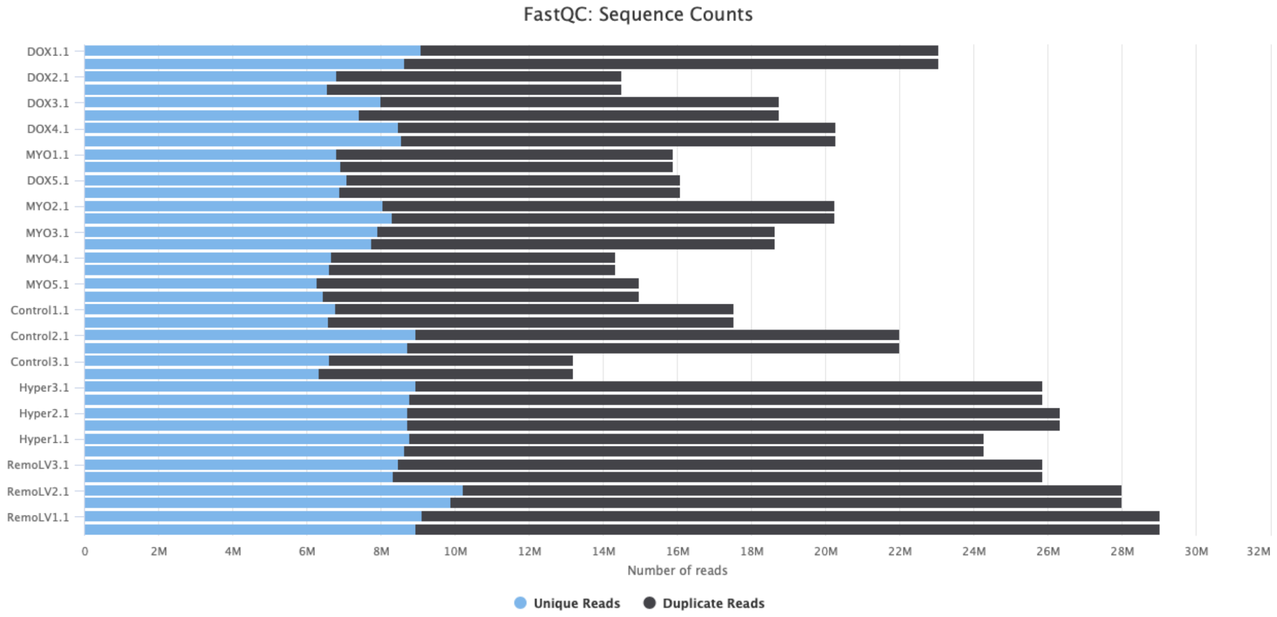

B

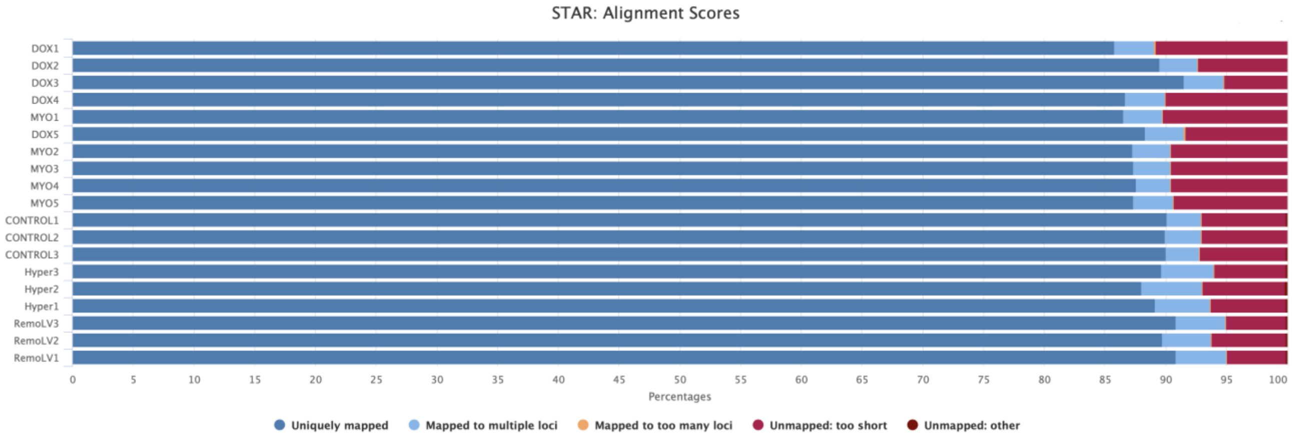

Supplementary Figure S2. Analysis and summary of RNA-seq data quality

Sequence counts for each sample with estimated duplicate read counts (A). Statistics generated after STAR alignment. Bars represent the alignment score (B).

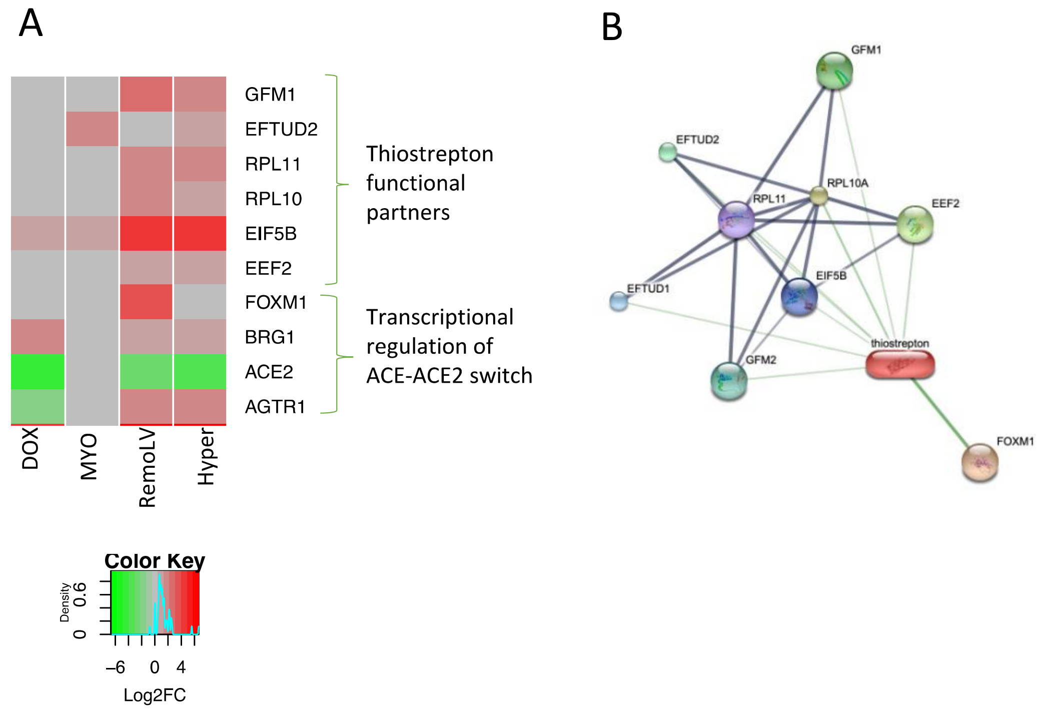

Supplementary Figure S3 Gene expression of Thiostrepton functional interaction partners involved in Angiotensin converting enzyme switch.

(A) Heatmap representing expression of selected genes in all treatment models DOX (N=5), MYO (N=5), RemoLV (N=3) and Hyper (N=3) compared to control group (N=3). (Red= upregulated, Green= downregulated, grey = non-significant). (**B)** Interaction networks of chemical perturbagen Thiostrepton. Protein-protein interactions are shown in grey, chemical-protein interactions in green and interactions between chemicals in red.
